# The Role of Targeted Microbiota Therapy in the Prevention and Management of Puerperal Mastitis

**DOI:** 10.3390/diseases13060176

**Published:** 2025-06-05

**Authors:** Mariarosaria Matera, Chiara Maria Palazzi, Alexander Bertuccioli, Francesco Di Pierro, Nicola Zerbinati, Massimiliano Cazzaniga, Aurora Gregoretti, Ilaria Cavecchia

**Affiliations:** 1 Department of Pediatric Emergencies, Misericordia Hospital, 58100 Grosseto, Italy; 2 Microbiota International Clinical Society, 10123 Torino, Italy; alexander.bertuccioli@uniurb.it (A.B.);; 3 Department of Biomolecular Sciences, University of Urbino Carlo Bo, 61029 Urbino, Italy; 4 Medicine and Technological Innovation Department, University of Insubria, 21100 Varese, Italy; 5 Scientific & Research Department, Velleja Research, 20125 Milano, Italy

**Keywords:** breastfeeding, dysbiosis, inflammation, mastitis

## Abstract

Mastitis, an inflammatory condition of the breast, significantly affects breastfeeding women and can lead to the early cessation of lactation. This article explores the pathophysiology of mastitis, distinguishing between acute mastitis (AM) and subacute mastitis (SAM), with a focus on the microbial dynamics involved. AM is primarily associated with *Staphylococcus aureus*, while SAM is linked to a dysbiotic milk microbiota characterized by an imbalance of microbial species, including increased levels of opportunistic pathogens. The role of inflammation and the gut–breast axis in the development of mastitis are discussed, emphasizing the importance of maintaining a healthy microbiota. Recent studies highlight the potential of probiotics as a preventive and therapeutic measure against mastitis, showing promising results in reducing incidence and recurrence. However, further research is necessary to optimize probiotic strains, dosages, and treatment protocols. This review underscores the need for a comprehensive understanding of the microbiological, immunological, and inflammatory factors involved in mastitis to develop effective prevention and treatment strategies.

## 1. Introduction

Breastfeeding is the gold standard for infant nutrition and short- and long-term health. Breastfeeding is closely associated with higher infant survival rates and significant health benefits for both infants and mothers, in a dose–response relationship [1].

The American Academy of Pediatrics (AAP) in agreement with the World Health Organization (WHO) recommends exclusive breastfeeding for the first six months of life and then continuing breastfeeding during the introduction of complementary foods for at least 24 months. Medical contraindications to breastfeeding are rare and include galactosemia, maternal HIV infection, or infection with human T-cell lymphotropic virus type I or II, untreated brucellosis or suspected or confirmed Ebola virus disease. Instead, the use of substances such as opioids, cocaine, and phencyclidine or alcohol, tobacco, maternal medications, and exposure to radiological procedures must be specifically evaluated since they can sometimes constitute contraindications to breastfeeding due to their potential effect on the long-term physical and/or neurobehavioral development of the infant [2].

Breast milk is a true liquid tissue that, in addition to its nutritional components, contains crucial bioactive components, such as stem cells, growth factors, microorganisms from the milk microbiota, and human milk oligosaccharides (HMOs), which play essential protective roles for both the infant and the mother [3,4]. Breast milk shapes the infant’s gut microbiota, promotes tolerance to commensals microorganisms, supports immune system maturation and neurocognitive development, protects against infections, and reduces infant mortality and morbidity [5]. The WHO recommends exclusive breastfeeding during the first 6 months and continued breastfeeding for up to 2 years [6]. Breastfeeding protects mothers from various health conditions, including cancers and type 2 diabetes [1].

For this reason, promoting and supporting the initiation, duration, and exclusivity of breastfeeding is a public health priority. However, global breastfeeding rates continue to fall short of international guidelines, and mastitis remains one of the factors that negatively impact breastfeeding [7].

Lactation mastitis is an inflammatory condition affecting breast tissue, with an incidence generally ranging from 2% to 33% [8,9,10,11] of women during the breastfeeding period. Different studies have reported varying prevalence rates, with incidences of 2.5% among breastfeeding women in the United States, 24% in Finland, and 27.1% in Australia. In a prospective study conducted on 946 breastfeeding U.S. women, 9.5% self-reported having lactation mastitis diagnosed by a healthcare provider within the first three months postpartum [12]. The condition presents with localized symptoms such as breast pain, engorgement, reddened, swollen, warm, and tender areas of the breast, along with high fever and flu-like symptoms, including muscle aches, chills, vomiting, fatigue, and general discomfort.

The etiology of mastitis is not yet fully agreed upon. It can result from inflammatory causes, infections, bacterial imbalances, or a combination of factors. There are cases in which mastitis can resolve without medical intervention [13,14]. International guidelines for treating mastitis recommend effective milk removal. This involves breastfeeding more frequently and, after each feeding, expressing excess milk manually or with a pump to promote recovery [13]. However, in some situations, depending on the duration and severity of symptoms [15], antibiotics may be required. This necessity, along with the associated pain, could lead to a premature cessation of breastfeeding due to the fear that the antibiotic might be transferred to the infant through breast milk [16]. Approximately 10% of women who develop mastitis stop breastfeeding. Regarding the progression to breast abscess, the literature is not entirely consistent on the incidence rates, which are reported to vary between 4.6% and 11% [17]. Meanwhile other works, such as that by Amir et al. [18], report lower rates. Such variability could be attributed to the heterogeneity of the study groups.

Breastfeeding is the primary source of nutrition for the infant and is associated with significant benefits for infant health; in addition to essential nutrients, immune cells, and bioactive components, breast milk also contains a wide range of microbes that support the development and stabilization of the infant microbiome and are important for maintaining breast and infant health [19]. Zimmermann et al. [20] conducted a review including 44 studies that analyzed 3105 breast milk samples from 2655 women. In human milk, 58 phyla, 133 classes, 263 orders, 596 families, 590 genera, 1300 species, and 3563 operational taxonomic units have been identified at the bacterial taxonomic level in addition to fungal, archaeal, eukaryotic, and viral DNA. The composition of the milk microbiota comes mainly from the mother’s intestine, partly from the newborn’s mouth, and partly from the peri-areolar skin, and is made up of multiple genera such as *Staphylococcus*, *Streptococcus*, *Lactobacillus*, *Pseudomonas*, *Bifidobacterium*, *Corynebacterium*, *Enterococcus*, *Acinetobacter*, *Rothia*, *Cutibacterium*, *Veillonella*, and *Bacteroides*. Some evidence suggests that the composition of the milk microbiota varies with gestational age, mode of delivery, biological sex, parity, antibiotics taken during delivery, stage of breastfeeding, diet, body mass index (BMI), composition of breast milk, HIV infection, geographic location, and method of collection and administration of milk to the infant. Fernandez et al. [21] explain that the milk microbiota is an essential component in guiding the stabilization of the infant’s intestinal microbiota. In the intestinal microbiota of the breastfed infant, infantile-type bifidobacteria (*B. longum infantis*, *B. logm longum*, *B. breve*, and *B. bifidum*) are predominant and colonize the newborn’s intestine due to their marked ability to metabolize HMOs [22]. Lordan C et al. [23] describe human milk oligosaccharides (HMOs) as a complex mixture of heterogeneous structures of complex and multifunctional glycans, widely represented in breast milk; these are not digested by the infant, therefore they remain completely intact in the infant’s intestine and play a prebiotic role for specific bifidobacteria called infantile-type, since they possess an adapted functional capacity to metabolize various HMO structures widely available to breastfed infants.

Antibiotic treatment can interfere with the process of microbiota structuring in the infant and with the proper maturation of their immune system. The use of antibiotics during breastfeeding affects the colonization rate of microorganisms in the infant, representing one of the earliest indicators of microbiota alteration. This could negatively impact the infant’s health, predisposing them to the development of allergies, neurological and behavioral disorders, and obesity [24]. Inflammatory conditions of the breast, such as mastitis, can also increase the risk of human immunodeficiency virus (HIV) transmission through breast milk, while exclusive breastfeeding may reduce this risk thanks to the protective properties of breast milk and its support for the infant’s immune integrity [25]. For all these reasons, preventing mastitis becomes a central goal for the health of the mother–infant dyad.

In 2020, Maree A. Crepinsek et al. [26] published a systematic review in the Cochrane Library analyzing ten randomized clinical trials involving 3034 breastfeeding women, aimed at evaluating preventive treatments for puerperal mastitis. Three studies ([27,28,29]) with 1038 participants compared the efficacy of probiotics to placebo, highlighting a possible reduction in the risk of mastitis with probiotics (relative risk [RR] 0.51; 95% confidence interval [CI] 0.35–0.75; two studies; 399 women), although the evidence was considered of low certainty. No adverse effects were reported, but the impact of probiotics on breast pain and nipple damage remains uncertain due to the low quality of available evidence. Moreover, the most extensive data from one study involving 639 women were not made public due to a lack of agreement between the researchers and the probiotic supplier, rendering the evidence incomplete. Subsequently, in 2022, Qinghoung Yu et al. [30] conducted a meta-analysis of six randomized controlled trials which confirmed that oral probiotic supplementation during pregnancy can significantly reduce the incidence of mastitis (RR 0.49; 95% CI 0.35–0.69; *p* < 0.0001) (Table 1). These findings suggest a potential role for probiotics in the prevention of mastitis during breastfeeding, although further high-quality multicenter clinical trials are needed to confirm these results.

In this review, we will examine the pathophysiological mechanisms of puerperal mastitis and their relationship with milk microbiota and the intestinal and oral inflammatory state of the breastfeeding woman. Finally, we will explore the potential prophylactic role of probiotics in preventing this inflammatory condition.

## 2. Factors Involved in the Development of Puerperal Mastitis

Although the pathophysiology of lactation mastitis is not yet fully understood, several factors may contribute to its onset, including excessive milk production, nipple damage, improper use of breast pump flanges, intense suction, dysbiosis, and abrupt or partial weaning. Specifically, milk overproduction can lead to persistent breast engorgement which, if not adequately drained, may progress to inflammatory mastitis or, in some cases, bacterial proliferation within the milk ducts (bacterial mastitis) [15].

Additional predisposing factors include first-time motherhood (primiparity), obesity, smoking, maternal malnutrition, health issues affecting the mother or infant, and improper breastfeeding positioning [35].

A review by Wilson et al. [16], based on 26 studies, identified the major risk factors for lactation mastitis. Among these, nipple damage—reported in 42% of the studies analyzed—was particularly significant, as it frequently occurs during the early postpartum period and provides an entry point for pathogens [36]. The CASTLE study (Candida and Staphylococcus Transmission: Longitudinal Evaluation) further highlighted the presence of *Staphylococcus aureus* on the nipple or in breast milk as a notable risk factor [36]. Although *Staphylococcus* species are among the predominant commensal bacteria in the breast milk of healthy women, their concentrations are significantly higher in breast milk samples taken from mothers with lactational infectious mastitis (LIM) [37].

In summary, epidemiological observations reported in the literature categorize the key risk factors for lactation mastitis as follows:

1. **Pathogen-Related Factors**: These factors include virulence factors, superantigens, biofilm formation, and the expression of antimicrobial resistance genes. *Staphylococcus aureus*, particularly its methicillin-resistant variant (MRSA), is one of the main pathogens responsible for skin and soft tissue infections, including mastitis [38,39]. Among the most relevant pathogens, *Staphylococcus aureus* [40] and coagulase-negative *Staphylococcus* together account for approximately 78% of mastitis cases [41]. These include streptococci and other Gram-positive and Gram-negative bacteria, as well as mycoplasmas [42]. *Staphylococcus epidermidis* is also one of the main causative microorganisms of lactational mastitis [8].
2. **Perinatal Factors**: Several research groups have analyzed the use of breastfeeding products as a potential risk factor for mastitis. While a positive association has been found between the use of various nipple creams and the development of mastitis during breastfeeding, it is not always clear whether the use of these products preceded or followed the onset of mastitis, or whether their use was triggered by other potential risk factors, such as cracked nipples [16]. Evidence linking breastfeeding frequency, positioning, milk production, and mastitis is also inconsistent and not well defined [16]. Moreover, women who have previously experienced mastitis during breastfeeding may be more prone to recurrent episodes in the future [41]. Improper attachment, which can lead to nipple trauma, may also contribute to the development of mastitis [36].
  The administration of antibiotics during the third trimester of pregnancy, childbirth, and/or breastfeeding could promote the development of infectious mastitis [43]. Antibiotic therapy, whether administered during cesarean delivery or as intrapartum prophylaxis for PROM (premature rupture of membranes), P-PROM (preterm premature rupture of membranes), or for Streptococcus agalactiae colonization, represents a significant risk factor. Antibiotic treatment can favor the selection of antibiotic-resistant staphylococci within the mammary gland, while simultaneously eliminating commensal strains that normally serve as natural competitors [42,44,45]. While many antibiotics, such as beta-lactams, macrolides, clindamycin, and Fosfomycin, are considered safe during pregnancy [46], they are known to have adverse effects on the microbiota present in human milk [8]. This, along with the growing resistance of *Staphylococcus aureus* strains to penicillin, methicillin (MRSA), and oxacillin (ORSA), could contribute to the failure of antibiotic therapy in the treatment of mastitis and its recurrence in mothers [8]. Moreover, the resistance to various antibiotics and the increased ability of *S. epidermidis* to form biofilms may explain the chronic and recurrent nature of this infectious condition [47].
3. **Host-Related Factors and Lifestyle**: Ethnicity, genetic background, breast structure and immunology, age (Women under the age of 21 and over the age of 35 seem to have a lower incidence) [8], number of lactations, and the phase of lactation are important. The anatomical structure and shape of the breast also appear to contribute, with the outer quadrants of the breast being more frequently affected [12]. Moreover, women with breast implants may have an increased risk of developing lactational mastitis within the first six months postpartum [48]. Cesarean delivery, compared to natural childbirth, appears to be associated with a higher likelihood of developing mastitis [49,50]. Cigarette smoking could contribute to the development of mastitis due to its harmful effects on the body, including reduced milk production, inhibition of the milk ejection reflex, inflammation, and impaired immune system function [51]. However, this association should be further investigated in more depth.
4. **Nutritional Status**: A reduced intake of several minerals (potassium, magnesium, phosphorus, calcium, manganese, and selenium) is associated with subclinical mastitis. Magnesium plays an important role in the immune system. Studies in mice have shown that short-term magnesium-deficient diets lead to an increase in pro-inflammatory cytokines and a reduction in bifidobacteria in the gut [52]. Selenium and vitamin E supplementation have been shown to reduce the risk of disease such as mastitis in periparturient dairy cows, due to their antioxidant role [53]. Selenium also helps reduce oxidative stress and enhance the immune system’s ability in cattle to respond to pathogens [54]. Vitamins C and E, β-carotene, and various B vitamins contribute to containing oxidative stress and the inflammatory response and promoting the functional homeostasis of the immune system. However, the use of these vitamins for the prevention or treatment of subclinical mastitis (SCM) is currently unclear. In women infected with HIV, the intake of vitamin A and β-carotene led to a higher risk of severe SCM [55], while studies conducted on vitamin E supplementation have shown more positive results suggesting a protective role for SCM [56]. Recent in vitro work has demonstrated the role of vitamin C as a direct growth inhibitor of *Staphylococcus aureus* often involved in the infectious progression of mastitis [57]. Afeiche MC et al. [58], for the first time, studied the association between the dietary inflammatory index (DII) and SCM in women. SCM was significantly associated with both DII and dietary micronutrient intake. They conclude the role of anti-inflammatory nutrients in reducing the risk of SCM should be evaluated in future studies with a larger sample size. Proper nutrition, which reduces maternal morbidity, can also help prevent mastitis, as nutritional deficiencies impair immune response and increase the risk of local inflammations [59]. Similarly, in dairy cows, metabolic disorders during the transition period can impair immune function and increase the risk of mastitis, once again emphasizing the importance of proper nutritional management [60].
  The nutritional status and the composition of the host’s gastrointestinal microbiome can impact the onset and development of mastitis. Rumen microbiota dysbiosis increases intestinal permeability, promotes the translocation of LPS into the blood, and contributes to the development of mastitis in cows [61].
5. **Socioeconomic Factors**: In the few epidemiological studies conducted on mastitis in North America, Australia, and New Zealand, higher household income, full-time employment, and higher education levels were associated with increased reporting of puerperal mastitis. This likely reflects not a higher incidence of the condition in these women, but rather greater awareness and the ability to report the issue to healthcare providers. However Yin et al. [62], in a comprehensive evaluation of the risk of lactational mastitis in Chinese women, did not find an education-related significant difference in terms of past medical history. The study by Hao et al. [63] highlights how socioeconomic factors, education, and healthcare support influence breastfeeding, indirectly reducing the risk of mastitis through correct and sustained practices. A recent review highlighted how informal employment negatively impacts the health of women and children, with conflicting results on breastfeeding, influenced by socio-economic and contextual factors such as maternity leave, work flexibility, and access to healthcare services [64]. Grzeskowiak LE et al. [65] studied the incidence of mastitis and antibiotic treatment in the first 6 months postpartum in 79,985 mother–infant dyads in the Norwegian Mother, Father and Child Cohort Study (MoBa). The study found that mastitis is associated with worse postpartum mental health. Lin CH et al. [66] conducted a population-based retrospective study in Taiwan during 2008–2017. Multivariable logistic regression revealed that multiparous women with a history of lactational mastitis within 6 months of giving birth were significantly more likely than nuliparous women to experience mastitis again after subsequent deliveries. Zarshenas M et al. [67] studied the incidence and risk factors of acute mastitis in the first 26 weeks postpartum in a cohort of Iranian women between June 2014 and March 2015. The incidence and risk factors were found to be similar to that reported for women in Western countries [12,42,68,69,70,71]. It is possible that women who are busy with work and family obligations, and therefore stressed, may be more likely to skip, delay, or reduce a meal, or supplement with formula milk, which can lead to blocked ducts. It is also likely that women in low-income settings have different risk factors compared to women in high-income settings, including access to breastfeeding products (e.g., nipple shields and breast pumps). Socioeconomic factors, such as maternal education and access to private or public healthcare, seem to influence the duration of breastfeeding and the incidence of mastitis. Women receiving private care appear to have a higher risk of developing the condition, possibly due to behaviors related to breastfeeding management, such as extending the intervals between feedings [72].

## 3. Pathophysiology of Mastitis

The pathogenic mechanisms of mastitis remain incompletely understood, and a universally accepted clinical definition is still lacking [16]. The definition of infectious disease has often been misapplied, as it has been used exclusively for cases involving *Staphylococcus aureus* [42]. According to medical literature, mastitis is an inflammation of the breast that may or may not be accompanied by a bacterial infection [73].

Some authors have suggested it might be more appropriate to classify mastitis into subgroups based on factors such as the stage of lactation (lactational or non-lactational); clinical presentation (clinical or subclinical); and progression (peracute, acute, subacute, or chronic) [42]. However, the concept of mastitis remains unclear due to evolving knowledge, differing etiological theories, and the variety of clinical symptoms [73]. The diagnosis of puerperal mastitis, also known as acute lactational mastitis, is primarily clinical [73]. It is characterized by the sudden onset of swelling, erythema, warmth, localized/unilateral pain, and systemic symptoms such as fever, chills, nausea, and vomiting.

*Staphylococcus aureus* is considered the primary etiological agent of acute mastitis (AM), often transmitted to the breast from the woman’s hands [16]. Although this bacterium is thought to be responsible for the aforementioned systemic symptoms through toxin production [8,74,75], systemic symptoms can also occur prior to infection, as when the alveoli become distended, the tight junctions between the lactocytes may allow large molecules to leak into the stroma, triggering inflammation and generalized symptoms [76,77,78].

It is interesting to note, however, that many milk samples from healthy women contain *S. aureus*, indicating it as a normal inhabitant of the skin and milk [36]. Many breastfeeding women have potentially pathogenic bacteria in their milk, but an increase in their number does not seem to affect the clinical onset of mastitis [79]. According to Ingman et al. [80], mastitis is influenced by the interaction between bacteria, the microbiome, and the immune response.

Subacute mastitis (SAM) is the most common form of mastitis among breastfeeding women and is the leading cause of unwanted cessation of lactation, with a significant impact on maternal and infant health [74]. SAM has a more insidious course with milder symptoms and does not correlate with a specific bacterial agent. The symptoms associated with mastitis result from the obstruction of the milk ducts and the impeded milk flow, leading to the progression of the inflammatory process in the breast tissue. Mammary inflammation appears to be associated with an imbalance in the microbial species of the milk microbiota, with an excessive growth of species capable of forming biofilms, which in turn obstruct the milk ducts, causing milk stasis, increased inflammation, and opportunities for opportunistic infections. Cultural investigations conducted on milk from women with SAM indicate a predominance of *Staphylococcus epidermidis*, a typical commensal of healthy skin and human milk microbiota, and a lower abundance of coagulase-negative staphylococci (CoNS) and *Streptococcus viridans* [74]. In milk samples with subacute mastitis, other species of *Staphylococcus* have also been detected, such as *S. hominis*, *S. pasteuri*, *S. warneri*, and *S. haemolyticus*, but in significantly lower quantities compared to *S. epidermidis* [8].

Several studies have shown that although mastitis positively correlates with an increase in the total bacterial count in milk, the severity of the condition is more closely related to the increase in inflammatory factors such as C-reactive protein, IL-1, IL-6, IL-8, and TNF-α, [42,74,76,81,82]. These observations have prompted recent studies using molecular methods, which have revealed that the same pathobiont microorganisms found in the milk of women with mastitis are also present in the milk of healthy women. In particular, at least five potentially pathogenic bacterial species have been identified in both SAM and healthy women’s milk: coagulase-negative staphylococci (CoNS), *Streptococcus viridans*, *Staphylococcus aureus*, group B streptococci, and *Enterococcus faecalis*. Additionally, although the presence of *S. aureus* and group B streptococci is more frequently observed in women with subacute mastitis (SAM) than in healthy women, it is important to note that about 30% of healthy women also have *S. aureus* in their milk, and 10% have group B streptococci [83]. This raises the question of why some women develop mastitis while others do not.

**Figure 1:**
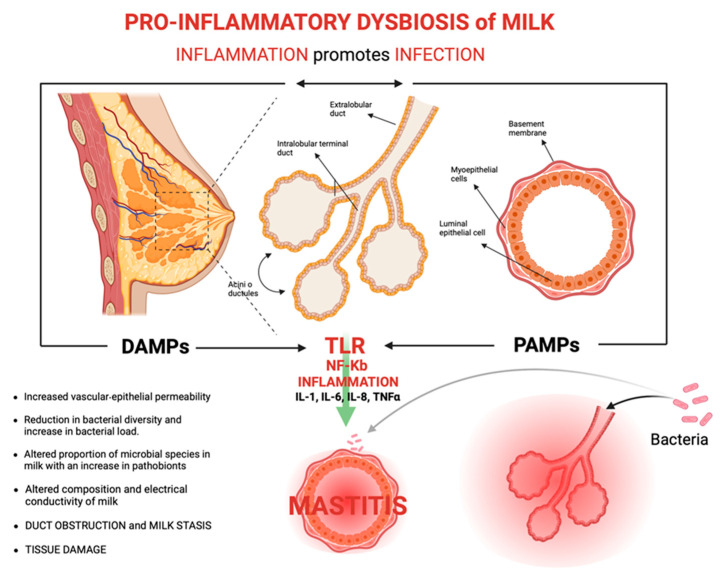
Pathophysiology of mastitis from a microbiome perspective. DAMPs: damage-associated molecular patterns; PAMPs: pathogen-associated molecular patterns; and TLR: Toll-like receptors.

We can make two considerations in this regard: the first is that the symptoms of mastitis are always associated with inflammation but not necessarily with the infection of the breast tissue, from which we deduce that mastitis is primarily an inflammatory pathology and that only in some cases can it complicate itself into bacterial mastitis. The second is that Toll-like receptors (TLRs) can be activated in case of infection by PAMPs (pathogen-associated molecular patterns), such as LPS, acids of the phospholipid wall, bacterial lipopeptides, and flagellin expressed by bacterial pathogenic microorganisms, but they can also be activated in the absence of infection by DAMPs (damage-associated molecular patterns), which include endogenous protein products expressed by host cells exposed to cell damage or death. This last condition is what could occur in the tissue inflammatory state that characterizes SAM. Both PAMPs and DAMPs, by binding to TLRs, activate the nuclear transcription factor NF-kB, triggering the transcription, translation, and release of inflammatory factors, chemokines, and adhesion molecules and the recruitment of the innate immune response with the amplification of the inflammatory state in the breast tissue (Figure 1) [76,82]. For further details on the virulence factors of the main bacteria involved in mastitis, please refer to Table 2.

To suggest the potential inflammatory mechanism in the pathogenesis of mastitis, it is interesting to point out recent studies conducted on electrical conductivity in breast tissue. In conditions of tissue homeostasis on the basolateral membranes of the epithelial cells of the milk ducts the sodium/potassium pump carries out an active transport of ions so that physiologically in the intracellular fluid the Na+/K+ ratio is approximately 1:3, while in the extracellular fluid it is approximately 3:1. The electrical potential gradient between the basolateral membrane and the lumen of the milk duct created by high intracellular levels of K+ and low levels of Na+ can be measured. The milk is electrically positive with respect to the epithelial cells of the milk duct and at the same time is isosmotic with respect to the plasma, since in the blood, as in milk, the Na+/K+ ratio is approximately 1:3. Differently, in the inflammatory state of the breast tissue, damage to the epithelial cells occurs with an opening of the tight junctions and an increase in capillary permeability so that higher levels of Na+ and Cl− enter the alveolar extracellular fluid of the mammary gland and, in order to maintain the osmotic pressure, the K+ concentration decreases. Furthermore, the persistence of the inflammatory state also leads to the damage and death of epithelial cells (lactocyte) with a reduction in the synthesis of lactose, which plays a crucial role in maintaining the osmolarity of milk so that it is isotonic with respect to plasma. The change in the electrical conductivity of milk detected during mastitis confirms the inflammatory mechanism. The inflammatory response determines an increase in epithelial and vascular permeability and parenchymal damage which leads to changes both in the composition of the milk in terms of lactose and in the electrolyte concentration and therefore in the electrical conductivity of the milk.

Studies carried out on animal models and confirmed in women have found, during the first stages of mastitis, even before the onset of symptoms, an increase in sodium and chlorine and reduction in potassium with the modification of electrical conductivity; these findings could therefore be used as screening for the early and specific detection of mastitis [76,82,87]. The second consideration we can make is with respect to the correlation between the characteristics of the microbiota of the mammary gland and milk, tissue inflammatory state, and the risk of developing mastitis. The milk microbiota physiologically has a bacterial count of less than 3 log 10 CFU/mL and the most represented bacterial species are staphylococci (in particular *Staphylococcus epidermidis*) and streptococci and, to a lesser extent, bifidobacteria and enterobacteria.

The mammary gland microbiota is a secondary microbiota and originates from the intestinal microbiota, the periareolar skin microbiota, and the oral microbiota and is mainly composed of Proteobacteria followed by Firmicutes, while the most abundant taxa are *Enterobacteriaceae*, Bacillus, *Pseudomonas*, *Staphylococcus,* and *Propionibacterium*. During pregnancy, starting from the third trimester, hormonal participation determines a physiological increase in intestinal permeability and blood–milk permeability. Due to increased intestinal permeability, dendritic cells transfer microorganisms from the maternal intestinal lumen to the mammary gland through the enteromammary circulation [21], while the increase in milk–blood permeability allows the transfer of bacteria from the mother’s oral cavity to the mammary gland, helping to enrich and build the milk microbiota through the oro-mammary circuit [5].

In 2019 Moossavi et al. [88] detected oral bacteria in expressed breast milk, suggesting possible bacterial transfer from the mother’s mouth to the mammary gland. Other studies propose that retrograde milk flow during infant sucking may allow bacterial transfer from the infant’s mouth to the breast [89,90,91].

**Figure 2:**
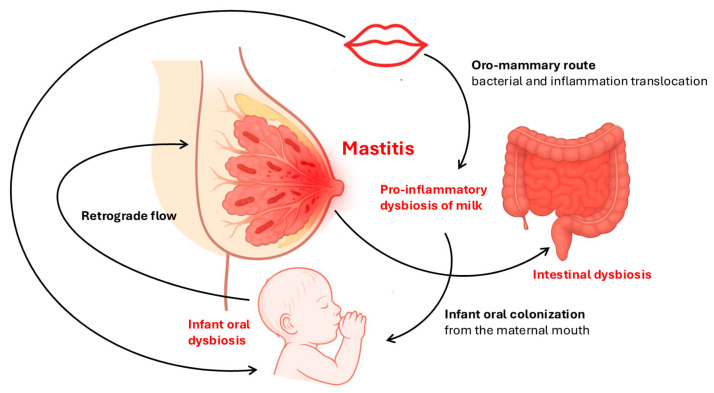
Milk dysbiosis as the origin of the risk of developing mastitis.

In physiological conditions, the bacterial strains present in the milk microbiota are in complete balance and it is precisely the commensal strains which, through competitive exclusion mechanisms and the production of antimicrobial compounds, such as bacteriocins, organic acids, or hydrogen peroxide, perform the task of inhibiting excessive growth of pathogenic bacteria, including those potentially capable of developing bacterial mastitis. It seems clear, therefore, that the eubiotic mammary microbiota might play a central role in the maintenance of mammary homeostasis, probably also with respect to the prevention of mastitis. The balance of the milk microbiota reflects the trend of the microbiota of the intestine, mouth, and skin of the woman; therefore, all dietary and lifestyle modifications, the possible use of antibiotics, and above all intestinal and oral inflammatory aspects correlate with risk, also pro-inflammatory at the breast tissue level, and therefore the risk of developing mastitis. The use of antibiotics, although essential for treating bacterial infections, can have a negative impact on the intestinal microbiota, disrupting its balance and promoting the development of intestinal dysbiosis. This condition compromises the body’s natural immune defenses, facilitating the proliferation of pathogenic bacteria and increasing susceptibility to infections, as well as mastitis. For example, the combined administration of meropenem, gentamicin, and vancomycin in adults leads to an increase in the prevalence of Enterobacteriaceae and other pathobionts, associated with a reduction in Bifidobacterium and butyrate-producing species [92]. Treatment with commonly used antibiotics, such as fluoroquinolones, cephalosporins, and metronidazole, can also significantly disrupt the intestinal microbiome balance, promoting the growth of Enterococci and *Candida* spp., while reducing the presence of *Bifidobacteria* and *Clostridia* [93]. Even though short, antibiotic treatment may lead to changes in the gut microbiota, such as reduced microbial richness and an increase in global Gram-negative bacteria. However, it is important to highlight how certain probiotic strains can be helpful in counteracting the negative effects of antibiotics on the gut microbiota. For example, *Bifidobacterium longum* W11 administered together with rifaximin has prevented damage to the gut bacterial consortium [94]. Moreover, the use of prebiotic substances could also prove to be a valuable ally in maintaining intestinal microbial balance [95]. Therefore, a prophylactic intervention, aimed at containing the oral and intestinal inflammatory state of the nurse, will also result in the prevention of mastitis (Figure 2) [42].

## 4. Milk, Microbiota, and Mastitis

Knowledge about the microbiology of mastitis is still limited. Boix-Amorós A et al. [74] studied the bacterial composition of milk from SAM women, aimed at characterizing the etiology of the disease and finding its potential bacterial biomarker. Despite its high prevalence, this condition is often underestimated and underdiagnosed. As mentioned earlier, the symptoms are generally milder; however, the formation of biofilms within the ducts, resulting from the overgrowth of commensal bacterial species such as CoNS and viridans streptococci, can inflame the mammary epithelium, causing a characteristic pain like a needle prick. Even though these ‘commensal’ bacteria are found in high concentrations in breast milk, the case is generally classified as non-infectious mastitis [75]. The authors carried out 16S rRNA sequencing on milk samples from healthy women and from SAM women in the symptomatic phase and when symptoms disappeared, and the bacterial load was significantly higher in the SAM milk samples in the symptomatic phase. However, bacterial diversity was reduced in the samples collected in the symptomatic phase and the authors did not detect a microbial species correlated to SAM since the microbial species found were the same even in healthy women; however, they detected a significant modification in the proportions between microbial species, indicating a dysbiotic ecological shift in SAM women compared to healthy ones. Finally, by exposing breast epithelial cell lines to SAM milk bacterial pellets, they detected the ability to stimulate an overproduction of IL-8 and support the inflammatory process of breast tissue. The authors therefore concluded that there is no specific causal agent in SAM, whose etiology is instead polymicrobial and variable and consequent to a pre-existing milk dysbiosis rather than the outcome of an external environmental infection. Despite the high interindividual bacterial variability in milk, specific differences have been identified between SAM women and healthy women: in SAM women in the symptomatic phase, an increase in anaerobic and opportunistic pathobionts such as *Finegoldia* and *Peptoniphilus* was observed, as well as *Corynebacterium kroppenstedti* (already isolated in previous works from samples of granulomatous mastitis and breast abscesses [96,97]), *S. aureus,* and oral species such as *Prevotella nanceiensis*. The RNA analysis also showed a particular association with the presence of *Lactobacillus iners* (a species producing the toxin innerylysin which can cause cell damage), *Neisseria subfava*, *Streptococcus lactarius,* and *Streptococcus cristatus*. Both in SAM women in the symptomatic phase and at the resolution of symptoms, *Pseudomonas* spp. and *Acinetobacter* spp. were decreased compared to healthy controls, while in SAM women upon the resolution of symptoms, there was an enrichment in oral commensals such as Streptococci, *Porphyromonas,* and *Prevotella* and in *Porphyromonas endodontalis* and *Streptococcus peroris*, and in *Acinetobacter johnsonii*, *Propionibacterium acnes*, *Lactobacillus helveticus,* and *Lactobacillus zeae*. Interestingly, *P. acnes* is a characteristic bacterium of the skin microbiome that displays anti-S auraus activity in vitro [98,99]. The presence of *Acinetobacter johnsonii*, *Corynebacterium simulans,* and *Acinetobacter lwofii*, *Propionilbacterium acnes*, *Staphylococcus hominis,* and *Lactobacillus helveticus* were found to be biomarkers of breast health in the milk of healthy women. The authors detected the presence of *Staphylococcus aureus* in both healthy and SAM women; however, *S. aureus* appeared to be more active in SAM women in the symptomatic phase.

The presence of *Streptococcus lactarius* was detected in 13 out of 23 milk samples from healthy women, but that detected in SAM women was significantly more active both during the symptomatic phase and at the cessation of symptoms. All this suggests that a modification in the proportion of microbial species in milk can favor opportunistic pathogens, trigger and/or favor the inflammatory process, and give rise to mastitis and its symptoms.

## 5. Gut–Breast Axis, Mouth–Breast Axis, and Retrograde Flow

Moossavi et al. [88] studied the influence of maternal, infant, and environmental factors on milk microbiota composition using redundancy analysis and identified several factors, including breastfeeding mode, lactation stage, and maternal BMI, to be associated with the overall milk microbiota community composition, although with very low redundancy values (each accounting for <2% of the variation in milk microbiota). The cumulative association of all the factors studied through a multivariate analysis explained approximately 25% of the total variation in milk microbiota composition. Host-related factors explain only a fraction of the microbiota composition, suggesting that factors shaping these microbial communities may also involve host genetics and immunity.

If we want to identify signs of prophylactic microbiota-targeted intervention with respect to mastitis, we must focus on understanding the origins of milk dysbiosis. The existence of an intestine–mammary gland axis has been described which allows, through the enteromammary pathway, the transfer of commensal bacteria from the maternal intestine to the mammary gland [88]. In the third trimester of pregnancy, the physiological hormonal movements, through the reduction in *Faecalibacterium prausnitzii*, promote an increase in intestinal permeability, thus carrying out the transfer of microbes and immune cells from the intestine to the mammary gland [100]. A mouth–breast axis has also been described, which allows microbial and cytokine transfer, via blood, from the maternal oral cavity to the mammary gland [88]. Finally, with the birth of the baby and the start of breastfeeding, sucking triggers a physiological microbial exchange between the infant’s mouth and the mammary gland called retrograde flow which participates in the competitive selection of the microbial species specific to the milk microbiota [30,101,102]. Hu et al. studied, on a mouse model, the regulatory mechanisms exerted by the intestinal microbiota with respect to the risk of mastitis caused by *Staphylococcus aureus* infection [40]. They found that in mice with intestinal dysbiosis, mastitis induced by *S. aureus* is associated with an increase in the permeability of the intestinal barrier and the blood–milk barrier, which makes the translocation of intestinal bacteria into the systemic circulation easier. Fecal microbiota transplantation (FMT) in dysbiotic mice reduced the severity of *S. aureus*-induced mastitis by restoring commensal homeostasis, SCFAs production, and containment capabilities of the blood–milk barrier, and therefore essentially reducing the infiltration of inflammatory breast tissue. Ma et al. [100] studied bovine mastitis and confirmed that the intestinal microbiota dysbiotic can contribute to mastitis.

Researchers observed that transplanting fecal microbiota from cows with mastitis into germ-free mice led to the development of mastitis symptoms in the mice, along with increased inflammatory markers. However, when probiotics were administered concurrently with the fecal microbiota transplant, the severity of mastitis symptoms and inflammation in the mice was significantly reduced. Therefore, the authors conclude that intestinal dysbiosis participates in the pathophysiology of mastitis and that probiotics may represent an effective and safe strategy to treat and prevent mastitis [100].

However, we must not overlook the other two constituent components of the milk microbiota: the oral component coming from the maternal oro-mammary route and the retrograde flow of microorganisms coming from the infant’s mouth. The hormonal changes that characterize pregnancy and breastfeeding promote a pro-inflammatory microbial settlement in the mouth of the pregnant and breastfeeding woman, with an associated increase in blood–milk permeability, which facilitates the translocation of bacteria and inflammation to the breast via the bloodstream.

## 6. Future Prospects and Possible New Probiotics to Be Tested in the Prevention of Puerperal Mastitis

The studies conducted so far on the prevention of puerperal mastitis would still be very limited and extremely heterogeneous in terms of the choice of probiotic strains, dosages, and timing of administration. However, the existing evidence would seem encouraging, and probiotics could represent an opportunity in the prevention and treatment of mastitis [103]. Similarly to what would have been observed in breast cancer, where an alteration of the gut microbiota might contribute to disease development and the use of probiotics and prebiotics would represent a promising strategy to support conventional therapies [104], in mastitis as well, microbiota modulation would emerge as a potential therapeutic approach. 

The considerations made regarding the microbiomic pathophysiology of mastitis would give rise to hope for the future importance of testing the role of intestinal commensal probiotic strains with anti-inflammatory activity and capable of counteracting the excessive permeability of the intestinal barrier and the blood–milk barrier. In this regard, the role of *Clostridium butyricum* CBM588 could be investigated. It is an obligate anaerobic Gram-positive bacillary probiotic, isolated in Japan, capable of producing various beneficial substances: enzymes, vitamins, small peptides, and above all butyrate. It is used for its anti-inflammatory, immunomodulatory, and antineoplastic role, and its excellent ability as an intestinal colonizer has been demonstrated, where it creates a microenvironment unfavorable to pathogens, inhibiting their epithelial attack. The ability of CBM588 to produce butyrate could contribute to modulating intestinal permeability and the persistent systemic inflammatory state, which would also be a prelude to the development of mastitis [105,106,107,108].

It could also be interesting to investigate the role of pasteurized strains of *Akkermansia muciniphila*. This is a Gram-negative, strictly anaerobic bacterium belonging to the Verrucomicrobia phylum, first isolated in 2004 from the feces of healthy subjects. Among its activities, it plays the role of mucus-degrader and at the same time stimulates goblet cells to produce new mucin, thus promoting the turnover of the mucosal layer. The role of one of its outer membrane proteins, Amuc 1100 (also active after pasteurization), would also be known, capable of interacting with Toll-like receptor 2 (TLR2), increasing the expression of genes coding for tight junction proteins and reducing intestinal permeability [109,110].

The maternal oral microbiota is also the constitutive source of the infant’s oral microbiota, which in turn participates in the constitution of the milk microbiota. It is therefore clear that, if we want to prevent mastitis, we cannot ignore the treatment of oral eubiosis in pregnant and breastfeeding women. An imbalanced oral microbiota has been linked to the development of periodontal diseases [111]. In this sense, it would make sense to test the role of well-characterized strains of *Streptococcus salivarius* such as strains K12, M18, and 24SMB, which could spatially compete with oral inflammatory species [112,113,114]. *Streptococcus salivarius* K12 is an oral commensal [115] known for the ability to produce two bacteriocins, Salivaricin A and B [116,117], with specific bactericidal activity directed against *Streptococcus pyogenes*, *Streptococcus pneumoniae*, *Streptococcus agalactiae*, *Morexella catarrhalis*, and *Haemophilus influenzae*, which is widely used in the prophylaxis of recurrent episodes of streptococcal pharyngotonsillitis and acute otitis media [118,119,120,121,122,123,124,125]. Streptococcus salivarius K12 also appears to play a significant role in the management of halitosis [126,127]. Furthermore, evidence suggests that S. salivarius K12 may inhibit the invasion process of *Candida albicans* [128,129]. This strain has also been shown to inhibit the production of IL-6 and IL-8 by gingival fibroblasts activated by periodontal pathogens [130]. It should also not be underestimated that K12 is able to inhibit the biofilm of staphylococci, and this could represent a further advantage in the anti-mastitis perspective. Its use is considered safe for humans [131,132]. *Streptococcus salivarius* M18 would also exhibit probiotic properties specifically supporting dental and oral health [133]. It is recognized for its ability to produce certain bacteriocins such as Salivaricin M active against *Streptococcus mutans* (responsible for the cariogenic process) and the Salivaricin A2, 9, M, and MPS active against *Actinomyces*, streptococci, staphylococci, *Listeria*, *Haemophylus,* and *Enterococcus*. And finally, *Streptococcus salivarius* 24SMB which however does not show activity on *Streptococcus pyogenes* as it does not possess genes for bacteriocins. However, it seems to be effective in treating recurrent respiratory infections in childhood [134] and could also have activity against the pathogens of acute otitis media (AOM) in children prone to otitis [135].

### Probiotics and Mastitis

The first studies on the use of probiotics in mastitis were conducted in vitro in bovine mastitis, and it emerged that the choice of lactobacilli strains can stimulate the immune response in cows and inhibit the formation of *Staphylococcus aureus* biofilm [136,137].

As regards studies on probiotic use in human puerperal mastitis, we mention Barker M et al. [103] who conducted a literature review from 2000 to 2022 and Yu Q et al. [30] who performed a meta-analysis that included six randomized controlled trials, finding that the administration of oral probiotics during pregnancy can reduce the incidence of mastitis (RR: 0.49, 95% CI: 0.35 to 0.69; *p* < 0.0001). Espinosa-Martos et al. [138] conducted a study aimed at identifying microbiological, biochemical, and/or immunological biomarkers of the effect of probiotic administration in mastitis. They enrolled 23 women with symptoms of mastitis and 8 asymptomatic women who were administered a daily dose of *Lactobacillus salivarius* PS2 10^9^ CFU for 21 days. Milk, blood, and urine samples collected before and after the probiotic intervention were screened for a broad spectrum of microbiological, biochemical, and immunological parameters. It emerged that the intake of *L. salivarius* PS2 in the mastitis group led to a reduction in bacterial counts and IL-8 levels in milk and leukocyte counts in milk and blood, and to an increase in IgE levels and IgG, epidermal growth factor (EGF), and transforming growth factor-β (TGF-β2) levels in breast milk. The trial by Bond D et al. [27] on the efficacy of oral probiotic L. fermentum CECT5716 administration in the post-partum period for preventing mastitis offers an interesting investigative perspective, but the data have not yet been published. Karlson et al. [139] enrolled 57,134 Norwegian women participating in the Noerwegian Mother and Child Cohort Study to verify whether the intake of probiotic milk during the first half of pregnancy was associated with a reduction in breastfeeding complications and a lengthening of breastfeeding times. The intake of probiotic milk containing Lactobacillus acidophilus La-5 (La-5), Bifidobacterium lactis Bb12 (Bb12), and Lactobacillus rhamnosus GG (LGG) was associated with an increased risk of mastitis [adjusted odds ratio (aOR) 1.09, 95% confidence interval (CI) 1.02–1.16]; however, breastfeeding cessation (aOR 0.95, 95% CI 0.91–0.96) was less frequent in probiotic milk consumers than in non-consumers, suggesting that the relationship between probiotic milk consumption and mastitis was not causal but likely related to socioeconomic confounding factors. Therefore, further randomized controlled trials are needed to understand the relationship between specific probiotic intake and breastfeeding complications.

However, the authors themselves point out that the results appeared to be affected by socioeconomic factors.

Bousmaha-Marroki L et al. [140] studied the probiotic potential of thirteen strains of *Lacticaseibacillus rhamnosus* isolated from the gut microbiota of healthy breastfed infants using in vitro stimulation of human immune cells. The strains were evaluated for their β-hemolytic activity, antibiotic resistance, and antimicrobial activities against the *Staphylococcus* and *Streptococcus* strains that are most often involved in mastitis. None of the studied strains showed β-hemolytic activity, all showed non-transferable chromosomal resistance to ceftazidime, trimethoprim/sulfamethoxazole, vancomycin, and cefotaxime, and sensitivity to ampicillin, penicillin, tetracycline, rifampicin, erythromycin, chloramphenicol, and imipenem. Of the thirteen strains, four *L. rhamnosus* were selected for their broad antistaphylococcal spectrum: *L. rhamnosus* VR1-5 and *L. rhamnosus* VR3-1 for their ability to inhibit *S. aureus*, *S. epidermis* and *S. warneri*: *L. rhamnosus* CB9-2 and *L. rhamnosus* CB10-5 for the antagonistic effect against *S. aureus* and *S. epidermidis* strains. The immunomodulatory analysis of *L. rhamnosus* strains revealed that two strains exhibited significant anti-inflammatory potential, evidenced by a strong induction of IL-10 and weak secretion of pro-Th1 cytokines (IL-12 and IFN-γ), and in particular the *L. rhamnosus* CB9-2 strain combined a broad spectrum of antistaphylococcal activity and a promising anti-inflammatory profile; therefore this strain could represent a probiotic candidate for the management of puerperal mastitis. Further studies are certainly needed, with large RCTs with specific probiotic strains, well-defined administration times, and dosages and clinical evaluation of breast complications.

Jiménez et al. [34] explored the effect of oral administration of the probiotic *Lactobacillus salivarius* PS2 during late pregnancy and early lactation to prevent mastitis in breastfeeding women. The product was a probiotic supplement in sealed 1 g sachets, containing 1 × 10^9^ CFU of *L. salivarius* PS2, a strain isolated from the milk of a healthy woman, along with carrier materials. At the end of the 12-week treatment, women in the probiotic group were 59% less likely to develop mastitis compared to those in the placebo group. One limitation of this study is certainly the small sample size. The diagnostic criteria used differed from those of other similar studies, such as the one by Leónides Fernández et al. [28], which demonstrated that oral administration of *L. salivarius* PS2 during late pregnancy can be an effective method to prevent infectious mastitis.

Finally, Maldonado-Lobón JA et al. [33] conducted a double-blind, randomized controlled trial to test three different doses of *Lactobacillus fermentum* CECT5716 with respect to the reduction of *Staphylococcus* loads in the milk of women with acute mastitis. The women were randomized into 4 groups of which three received *Lactobacillus fermentum* CECT5716 for 3 weeks, respectively, at a dose of 3 × 10^9^ (CFU)/day, 6 × 109 CFU/day or 9 × 109 CFU/day, while the fourth group received a maltodextrin placebo. The authors found a significant decrease in the *Staphylococcus* load in the probiotic groups compared to the control group (*p* = 0.045). Furthermore, compared to the three different probiotic dosages, they detected a significant difference in the pain score of *p* = 0.035, *p* = 0.000 and *p* = 0.028, respectively, compared to the control group; however, they did not observe significant differences in terms of dose–response, obtaining similar results with all three probiotic dosages.

## 7. Conclusions

Recent studies allow us to hypothesize that probiotics, particularly *Lactobacillus* strains like *L. fermentum* CET5716 and *L. salivarius* PS2, could help prevent puerperal mastitis, reduce its recurrence, and promote healing. Probiotic supplementation during pregnancy and breastfeeding has shown promising results in improving breast health and modulating immune responses. However, further in-depth research is needed, including large-scale randomized trials, to define optimal dosages, timing of administration, and interactions with other treatments. Probiotics could become a key component in a more personalized approach to mastitis management, less dependent on antibiotics, benefiting both maternal and neonatal health.

## Figures and Tables

**Table 1:** Key Characteristics and Outcomes of Studies on Probiotic Interventions for Mammary Health. Adapted from Yu et al., 2022 [30].

First Author and Year	Participant Characteristics	Sample Size (N) EG/CG	Age (Years) EG/CG	Probiotic Strain	Dosage and Intervention Duration	Primary and Secondary Outcomes Investigated
[31]	Women with clinically diagnosed mastitis (bacterial count >4 log_10_ CFU/mL, leukocyte count >6 log_10_ cells/mL)	10/10	NR	*Lactobacillus salivarius* CECT5713 and *Lactobacillus gasseri* CECT5714	1 × 10^9^ CFU/day, -4 weeks	Bacterial count in breast milk; detection of the lactobacillus strains in milk samples
[32]	Women with mastitis, groups A, B, and C (specific clinical details not provided for all groups)	124(A)/127(B)/101(C)	NR	*Lactobacillus fermentum* CECT5716/*Lactobacillus salivarius* CECT5713	1 × 10^9^ CFU/day, 3	Breast pain; bacterial count in breast milk; adverse reaction; and detection of the lactobacillus strains in milk samples
[33]	Women with clinical mastitis (bacterial count >3 log_10_ CFU/mL); absence of abscesses or other mammary pathology	23(EG I)/24(EG II)/24(EG III)/27(CG)	33.3 ± 5.2 34.3 ± 4.3 36.0 ± 2.8 33.4 ± 4.5	*Lactobacillus fermentum* CECT 5716	3 × 10^9^ (EG)CFU/day, 6 × 10^9^ (EGI)CFU/day 9 × 10^9^ (EGII)CFU/day, 3	Breast pain; bacterial count in breast milk; adverse reaction; and immune parameters in breast milk
[28]	Healthy pregnant women (no probiotic supplementation or antibiotic treatment in the previous 30 days)	55/53	31.18 (±0.48)/ 30.51 (±0.49)	*Lactobacillus salivarius* PS2	1 × 10^9^ CFU/day, -8	Incidence of mastitis; breast pain; bacterial count in breast milk; and detection of the lactobacillus strains in milk samples
[29]	Healthy pregnant women who received prophylactic antibiotics (48 h before and after delivery)	139/152	31.91 (±0.49)/ 32.19 (±0.48)	*Lactobacillus fermentum*	3 × 10^9^ CFU/day, 16	Incidence of mastitis; breast pain; bacterial count in breast milk; and immune parameters in breast milk
[34]	Pregnant women ≥18 years of age, with intention to breastfeed	165/163	33.00 (±3.00)/ 33.00 (±3.00)	*Lactobacillus salivarius* PS2	1 × 10^9^ CFU/day, from the 35th week of pregnancy until week 12 after delivery	Incidence of mastitis; breast pain; and adverse reaction

**Table 2:** Virulence factors of the most important bacteria involved in mastitis (*Staphylococcus aureus* and Coagulase-negative Staphylococci (CoNS).

Virulence Factors	Coagulase-Negative Staphylococci CoNS	Coagulase-Negative Staphylococci CoNS	Coagulase-Negative Staphylococci CoNS	Coagulase-Negative Staphylococci CoNS		References
	*S. epidermidis*	*S. haemolyticus S. saprophyticus*	*S. capitis*	*S. lugdunensis*	*S. aureus*	
**TOXINS**	Metalloproteases Delta/Beta-hemolysins		Metalloprotease Hemolysine	Metalloprotease Coagulase δ-like-hemolysin	Panton-Valentine leucocidin (LukSF-PV) Epidermolysins (ETA-B-D) Hemolysine Superantigens Leukotoxin biocomponent pore-forming complexes (LukMF) Enterotoxins (*sea*, *seb*, *sec*, *sed*, *see*) Enterotoxin-like protein (*seg-sei*, *seij-seiq,* and *seiu*) genes Toxic shock syndrome toxin 1 (TSST-1)	[84,85]
**ADESION FACTORS**				Fibrinogen-binding protein	Capsular antigen polysaccharide/adhesin (PS/A)	[84,85]
**CAPSULE FACTORS**					X	[84]
**BIOFILM-ASSOCIATED PROTEINS**	Sequence IS256 and ica genes Phenol-soluble modulin (PSM) peptides Biofilm-associated proteins (bhp)		Phenol-soluble modulin (PSM) peptides			[84]
**CELL WALL-ANCHORED PROTEINS**	Adhesive matrix molecules (MSCRAMMs)	Adhesive matrix molecules (MSCRAMMs	Adhesive matrix molecules (MSCRAMMs	Adhesive matrix molecules (MSCRAMMs	Adhesive matrix molecules (MSCRAMMs) Iron-regulated surface proteins	[84]
**Virulence genes located on mobile** **genetic (pathogenicity islands, plasmids, and phage)**						[84]
**PROTEASE**	Serine protease Cystine protease Lipase Formate dehydrogenase (fdh) Metalloprotease	Lipase Autolysin Serine protease Cystine nuclease Urease	Lipase Cysteine protease Serine protease Nuclease			[84]
**ANTISEPTIN RESISTENCE GENES**	qacA ccrA ccrB IS256-like transposase gene				blaZ mecA mecC tetL tetK tet M tet0 ermA ermB ermC ermT msrA mphC InuA aacA aphD aphA3 mepA grlA gyrA	[84,86]
